# Two Memoirs on the Variations of Color of the Venous Blood

**Published:** 1859-05

**Authors:** Claude Bernard


### TRANSLATIONS FROM FOREIGN JOURNALS.

[From Brown-Sequard's " Journal de la Physiologie."]

### TWO MEMOIRS ON THE VARIATIONS OF COLOR OF THE VENOUS BLOOD.

BY PROFESSOR CLAUDE BERNARD.

[Read to the Academy of Sciences, Aug. 9 and Sept. 6, 1858.]

TRANSLATED BY THOMAS BEVAN.

*I. On the influence of the two orders of nerves which determine the variations of color of the venous blood in the glandular organs.*

In a communication, made to the January meeting for 1857, I showed that the glandular venous blood and the muscular venous blood presented an absolutely opposite color when considered during the active state of these organs.

When a muscle acts and contracts itself, the venous blood that is poured out is very dark. When the gland is secreting, and expels the product of its action, the venous blood it furnishes is, on the contrary, of a scarlet color, at times entirely identical with arterial blood. Whence it follows, in glands of intermittent secretion, there exists an alternate coloration of red and dark in the venous blood, according as the organ is in the one or other of the two physiological conditions, denominated states of function and repose.

After having established these preliminary facts, I continued my researches, with the purpose of determining what were the modifications of composition, corresponding to these so marked differences of color. I have succeeded, I believe, in finding the explanation. But, before entering upon the exposition of the experiments which relate to the purely chemical side of the phenomenon, I believe it indispensable to make known the physiological conditions of the nervous system, which regulate these special chemico-organic actions. I will even insist upon this subject, because the study of the mechanism by which the nerves act to effect the chemical phenomena which occur in the living organism, has always appeared to me the capital object which should pre-occupy the physiologist.

I desire to show to-day, that the particular chemical conditions which in the glands make the blood appear sometimes red, at others dark, are determined by the influence of two nerves, which have distinct origins, and possess in some sort, antagonistic action. In other words, that there exists a glandular nerve, which leaves the color of the venous blood red, and another which makes it become dark. I will show afterwards, that each of these nerves, to act chemically on the blood, modify, in an opposite manner, the mechanical phenomena of the capillary circulation; so that it establishes a necessary correlation, easy to be understood, between the chemical modifications the blood experiences in the organic tissues, and the mechanical conditions of the capillary circulation which are under the immediate influence of the nerves.

In order to the better understanding of the facts which are to follow, and for facilitating the study to those who wish to reproduce them, I ought to say, that all the results of the experiments here in question have been obtained upon the sub-maxillary gland of the dog, which is particularly proper for this sort of research, on account of its intermittent secretion, which renders very clear the variations in color of its venous blood.

II. The nerve that causes the red appearance of the venous blood in the vein of the sub-maxillary gland is a filament which detaches itself behind the lingual branch of the fifth pair. But it is only joined to the fifth pair; it in reality comes from the seventh, and is especially constituted by the *chorda tympanæ*. However it may be, this glandular-nervous filament may be easily reached at the point where it separates from the lingual to go to its distribution in the sub-maxillary gland, accompanying its excretory duct.

Now, when we consider the sub-maxillary gland provided with all these nerves, in a state of repose, that is to say, when nothing passes by its secretory duct, it is observed that the venous blood possesses a dark color. But, if at this moment the glandular nerve above mentioned is made to act, the blood which was previously flowing dark, becomes more and more red, and soon appears as entirely so as the arterial, and permits the establishment of this physiological proposition, that, when the action of the tympanico-lingual nerve is manifested energetically, the venous blood of the sub-maxillary gland appears red, while it becomes dark when this nervous filament fails to act, or when its action ceases to preponderate.

Nothing is more easy than to give the experimental proof of this special influence of the tympanico-lingual nerve on the color of the venous blood. In effect, when, after having exposed the glandular vein, and the nervous filament in question, we determine a gustative impression by putting a little vinegar in the mouth, we see the blood become rapidly ruddy in the vein, because the gustative impression produced on the tongue, and carried to the nervous centre, has been transmitted by reflex action through the chorda tympanæ. The proof of this interpretation is given immediately; for, if we cut the tympanico-lingual filament at the point where it separates from the lingual nerve, we observe the venous blood of the gland to remain dark, and frem this moment, notwithstanding the instillation of vinegar on the tongue, notwithstanding the gustative sensation perceived, the red color fails to re-appear, because the nervous route by which it received this modifying power on the blood has been obstructed. But if, taking this glandular nerve at the point of section behind the lingual, we irritate by means of galvanism its peripheral end, which still holds to the gland, we soon see, under the influence of this artificial excitant, the blood in the glandular vein become red, and again take its dark color when the excitation has ceased. This last experiment furnishes, therefore, a new argument to prove that the red color of the venous blood of the sub-maxillary gland is in direct relation with the activity cf the tympanico-lingual nerve, and that its dark color depends, on the contrary, on its state of physiological inactivity.

But it will not do to believe that, in case of repose of the gland, that the dark color of the blood seen is nothing but the passive result of the paralysis, or default of action of the tympanico-lingual nerve. This dark color of the blood is itself due to the action of another nerve, which acts in rendering the blood dark, and whose permanent influence is shown to be antagonistic to the tympanico-lingual, whose action appears to be more properly of an intermittent character.

III. The nerve which renders the venous blood dark in the sub-maxillary gland comes from the great sympathetic, and arrives in the gland accompanying the arterial branches of the external carotid, which go to it; the one small, penetrating the gland at its posterior and superior part; the other, the principal glandular artery, enters at the umbilicus of the gland by the side of its excretory duct. These glandular filaments of the sympathetic nerve come off, for the most part, from the superior cervical ganglion, they anastomose elsewhere, with filaments coming from other sources, and particularly the mylo-hyvidien, at the point where this nerve crosses the track of the facial artery.

When we consider the sub-maxillary gland in the physiological state, with all its nerves in repose, we have said the venous blood is dark; but that comes at the moment, from the activity of the great sympathetic, which renders the blood dark, predominating that of the tympanico-lingual, which renders it red. This is very easily proven; for, in this condition, if we cut all the filaments of the great sympathetic, which go to the gland, we see the venous blood lose its dark color to take a ruddy tint, which becomes permanent, because the nervous influence of the sympathetic is interrupted, and does not reach the gland.

But, if we now re-establish artificially the influence of this nerve, and excite by galvanism the peripheral end of the sympathetic filament, which still holds to the gland, we soon see the venous blood become very dark, to retake its red color when the galvanization of the nerve has ceased to act. We may, therefore, formula a physiological proposition inverse to that expressed for the tympanico-lingual, and say that the venous blood of the sub-maxillary gland is dark at all times, when the sympathetic acts, and is so in proportion as the action of this nerve is energetic.

By what precedes, we have acquired the experimental demonstration, that the variations of color of the glandular venous blood are due to two nervous influences, well determined and entirely distinct. But, how understand the mechanism of this influence of the nerves on the blood? There is no anatomical continuity, and, in consequence, no direct chemical action possible, on the part of the nerves upon the globules of blood, to modify their color. It is necessary, therefore, that there should be other intermediate phenomena between the nervous action and the chemical modification of the blood globule. In fact, these intermediate conditions exist, and are constituted by the diverse mechanical modifications which each nerve brings to bear in the capillary circulation of the gland, modifications which we will now examine.

IV. The mechanical conditions of the capillary circulation determined in the sub-maxillary gland by the tympanico-lingual nerve, and by the sympathetic, are exactly opposite.

When the tympanico-lingual nerve is excited, the venous blood appears red, and at the same time, there supervenes a considerable increase in the activity of the circulation. In proportion as the venous blood becomes redder, it circulates more and more readily, and the quantity which flows from the vein is shown to be much more considerable.

When the great sympathetic acts, it renders the venous blood dark, and at the same time lessens the activity of the circulation. The blood flows from the vein in less quantity, as it is more dark; even if the action of the sympathetic nerve is tolerably energetic, the sanguinous flow may be completely arrested in the vein, to reappear when the excitation of the sympathetic ceases, and to become accelerated anew, if the tympanico-lingual nerve is acted upon.

These results, which are constant, teach us, therefore, that the red and dark coloration of the venous blood is in a determined ratio to the rapidity of the circulation in the sub-maxillary gland. But this rapidity itself, of the course of the blood, cannot be effected by the nerves, which do not act, in any case, directly on the sanguinous fluid. The contraction and dilatation which we have seen to exist in the vessels of the gland can alone account for these modifications in the properties of the blood.

[To be continued.]

